# Supplementary material for: Optimizing immunization services: A Data Envelopment Analysis (DEA) of child immunization facilities in Pakistan
Source: PLoS One. 2024 Mar 22;19(3):e0298308. doi: 10.1371/journal.pone.0298308 (PMC10959375; doi:10.1371/journal.pone.0298308)
Supplement: Attachment — Submitted filename: Response to Reviewers.docx [file pone.0298308.s004.docx]

We thank the reviewers for thorough assessment of the manuscript and insightful comments. In some cases, we made few errors and accept the changes that were given by the reviewers. We hope and have addressed the comments in best of the our abilities in the table below.

| Reviewer | Response |
| --- | --- |
| **Reviewer 1 comments** | |
| 1. The abstract should be revised to ensure that it provides enough information for the reader to grasp the major issues discussed in the paper.  a. the abstract should provide clear and succinct information on the study context (location, study period), and the goal of the study.  b. It should specify what kind of models are being described. Describing DEA before discussing models could work.  c. The result section of the abstract should include details like the overjall numbers of health facilities included in the analysis.  d. The conclusion should focus on the policy or practical implications of the findings of the study. | Revised the abstract according to the comments in each section. |
| 2. The authors state that 22 facilities were dropped from the data. Facilities with missing observations were also dropped. It may be preferable to include these facilities in a sensitivity analysis to show what impact they have on the results. This is important given the lack of clarity on how facilities were randomized for inclusion, considering the acknowledgement that budgetary allocation influenced the extent of the survey. | In our analysis using STATA, facilities with missing inputs or outputs were excluded as DEA doesn't compute efficiency for such cases. Additionally, the decision to exclude 22 facilities with high outliers was based on logical considerations, especially in cases where stock used exceeded stock inputs by an illogical margin (50 to 200 percent higher), meaning huge error in numbers. Including such outliers could disproportionately impact the results and potentially skew the efficiency rankings. Hence, we are skeptical of using outliers in sensitivity analysis. |
| 3. The authors need to justify the choice of simple imputation for missing Penta 2 data. Why was multiple imputation which uses more variable not considered. The simple imputation resulted in a value for Penta 2 which was lower than both Penta 1 and 3. | The decision to use simple imputation was made based on the specific circumstances surrounding the dataset. Unfortunately, Penta 2 doses data were entirely missing, which posed a challenge for employing more sophisticated techniques like multiple imputation that typically require some observed values for imputation and follow trend on existing data. Implicating that the imputation technique does not assume on its own regarding the placement of Penta 2 doses between Penta 1 and Penta 3 doses. |
| 4. It is unclear how the authors made the decision to combine data across the years for Table 2. It will be useful for the reader to see the disaggregated data for each year. | Table 2 is updated. Disaggregated data has been added. |
| 5. It does not seem to me that the authors accounted for usual vaccine wastage rate in the analysis. This a common feature of vaccination programmes and need to be factored onto efficiency calculations. | We opted not to factor in the wastage of doses as it cannot be considered an input in the model. However, utilizing DEA has the advantage that each facility's efficiency is not treated as mutually exclusive. The efficiency of each facility is contingent upon the doses administered or used. The routine wastage rate of facilities is assumed to be adjusted when DEA assigns most efficient facilities, assuming uniform wastage across all facilities based on the vaccine type. |
| 6. The suggestion that the findings of this analyses may be the basis for redeployment of resources need to be tempered given that a lot of potentially important factors are not considered in this analysis. For instance, demand-side considerations, equity and access issues all go into resource allocation decisions in public health programs. Furthermore, it is unclear from the description of the data collection period if year-round data was collected for the entire study period to account for potential seasonality in trends. | The survey exclusively incorporated data from the facilities, focusing solely on supply-side factors. The primary objective is to assess whether resource optimization has been accomplished in immunization. However, the distribution of doses aligns with the catchment areas of the facilities. Equity and access issues are partially covered through target-oriented disbursement of the doses. This is mentioned in text line 188-191.  To mitigate seasonality trends, data spanning the past six months was considered. For the DEA analysis, we computed an average over six months to further diminish the potential impact of trends or errors within individual months." |
| **Other Comments** |  |
| 1. The flow of thoughts on the paper can be improved. The services of a language editor may be helpful with this. For instance, paragraph 2 of the introduction mentions a “recent grant from the Gavi…” without an indication of whom the grant recipient is. | This has been addressed briefly in the introduction. Text line 106-108, the complete information has been mentioned about the project. The study team for the project. |
| 2. The sentence in the last paragraph of the introduction that reads, “…last assessment fell in the COVID-19 period” was unclear to me. | The fourth survey was conducted in November 2020, which fell during COVID-19 pandemic. Hence, in the study we estimated the COVID-19 period’s effect on the efficiency scores of the facilities. |
| 3. A lot of the material in the discussion session on model specification can be moved to the strength and weakness section of the discussion. | It has been incorporated and added into the discussion section from text line 376 to 385. |
| 4. It is unclear to me what the numbers in brackets in the first paragraph of the results section mean. | In brackets we are showing the efficiency scores. However, we only mentioned the doses administered figures in the brackets. We have updated the numbers in the text. Text line 261 – 265. |
| **Reviewer 2** | |
| 1- Why do you use input-oriented DEA model is vague and needs more clarifions. Why not output-oriented one? | The comment has been addressed in text lines 188 to 191. An input-oriented model is employed, considering that immunization facilities receive a fixed, predefined set of vaccine stock. The stock is determined based on the catchment area of the facility, and other equipment is aligned accordingly. |
| 2- why do you use constant return to scale DEA model needs more explanations. why not variable return to scale one? | The comment has been addressed in text line 191 to 193: “The DEA was run with constant returns to scale, as all facilities are homogenous in nature. The equipment is similar and same level of staff is dedicated for immunization services across all facilities.” |
| 3- The validity of the proposed approach is vague. More analysis and discussion for the obtained results of the numerical example should be provided to prove the efficiency. | Our validity approach involved utilizing the variables of doses administered and Penta doses used. Despite their different natures, the extracted results demonstrate similar trends in efficiency across facilities, thereby validating our approach. However, we acknowledge that Penta used may be overstated, leading us to recommend the use of doses administered for a more accurate representation.  Moreover, we employed Tobit and Simar-Wilson regressions to cross-validate the impact of external variables on efficiency. Utilizing two model specifications and two regression techniques for external variables contributes significantly to enhancing the depth of our validity and sensitivity analysis. |
| 4- Author should update the introduction section deeply by stating the gaps of the existing study and then highlights the major results. | The concern has been addressed in text line 91 to 95. |
| 5- The literature review should be extended. Add and discuss papers with the application of the DEA in other fields. Please cite the following ones:  Equivalence relationship between the general combined-oriented CCR model and the weighted minimax MOLP formulation (2012) Journal of King Saud University-Science 24 (1), 47-54. An integrated data envelopment analysis and simulation method for group consensus ranking (2016) Mathematics and Computers in Simulation 119, 1-17. An interactive MOLP method for identifying target units in output-oriented DEA models: The NATO enlargement problem (2014) Measurement 52, 124-134. Fuzzy stochastic data envelopment analysis with application to NATO enlargement problem (2019) RAIRO - Operations Research 53 (2), 705721. A New Method for Solving Dual DEA Problems with Fuzzy Stochastic Data (2019) International Journal of Information Technology & Decision Making 18(1), 147-170. Fuzzy Stochastic Data Envelopment Analysis with Undesirable Outputs and its Application to Banking Industry (2018) International Journal of Fuzzy Systems 20 (2), 534–548. A hybrid DEA-MOLP model for public school assessment and closure decision in the City of Philadelphia (2018) Socio-Economic Planning Sciences 61, 70-89. | References have been cited as suggested, text line 89, 91 and 93. |
| **Reviewer 3** | |
| In the study, 4 different periods from 2018 to 2020 were discussed. For this reason, it was said that the effect of Covid was also examined. However, I see in table 3 that out of the 4 terms, only the second term has the lowest score. This already includes the period from May to October 2018. That's why the comments that it remained low due to the effect of Covid 19 are not very accurate. It should have been announced when there was a lower scoring period. I suggest changing the title. The study is not about Covid 19. | Revised as suggested. |
| Additionally, it is wrong, in my opinion, to suggest that DEA's potential improvement recommendation is to reduce the number of employees. Because the number of employees is an uncontrollable variable. I recommend that an analysis be carried out taking this issue into consideration. | We acknowledge the validity of the comment. While all inputs are necessary in the DEA model, we retained the staff members' variable. However, we emphasize that our primary and highlighted concern pertains to stock levels. Consequently, we have advocated for resource optimization specifically addressing the issue of overstocking, which, in absolute terms, emerges as the primary concern. |
| While defining the DEA model, the authors expressed both the objective function and the equality constraint as the entire DMU. However, this is not true. Only the DMU of interest should be included in the objective function and equality constraint. Therefore, the model is wrong and should be revised. | We have corrected the objective function and equality constraint in the manuscript, text lines 193 to 203. |
| While you should use the same number of data for both models, for example, we see that there are 93 units in one and 96 units in the other for time period t1. These should have been the same. What is the reason why it is different? This causes misinterpretation when comparing average scores with each other. | We decided not to keep the balanced panel for our analysis. The reason for dropping a few facilities is due to outliers and some missing data for either outputs and inputs, and these errors were at random during the survey. Making it a properly balanced panel would have made us lose more facilities. Using large sample was our priority. |
| Finally, the literature review should be expanded. Although the manuscript discussed many studies, it seems to be concentrated on a certain group of studies. | Literature has been extended in the introduction, paragraph 3 and 4, where we have added the application of DEA in different sectors and it’s utilization to estimate efficiency. |
| **Reviewer 4** | |
| Inputs and outputs should be more and better clarified. | Revised as suggested. Text lines 134 to 138. |
| English language should be polished. | The draft was revised to make sentences clearer and more concise. |
| In this study, it has been stated "the most efficient facilities are given a value of 1 (100%) whereas the least efficient facilities are given the value of 0 (or 0%); as the value drops from 1 (100%) to 0 (0%), technical efficiency of the facilities decreases." | The sentence has been revised in the text line 167 to 170.  The value of least efficient DMUs is close to zero, not zero. |
| Has the efficiency score been obtained equal to zero in the considered DEA model? |  |
| In this research, it has been expressed "The value of ℎ is censored between 0 and 1 as efficiency cannot exceed 100% and  cannot be non-positive." Does mean it can take zero?? | It is not indicating that efficiency can take the value of zero, rather just mention the range in which the |
| "u_r" ad "v_i" is correct. They should be investigated and revised.  Specify the units under review in detail. | We have revised the model. Text line 193 to 203. |
| Is the DEA model calculated for different years? Is not it better to use multi-period models? | We intended to consolidate all facilities into one pool to enable an assessment of efficiency across different time periods, gauging whether efficiency has shown an upward or downward trend over time. However, the analysis revealed that the efficiency levels remained relatively consistent across different time periods. |
| Why the VRS has not been used instead of the CRS assumption. | Because the facilities are homogenous in our model. Explanation has been added to the manuscript, text lines 191 to 193. |
| Future research directions should be improved; in that, they should stem from the awareness of the limitations and opening avenues related to the obtained outcomes. | The limitation section has been rewritten to include all opening avenues for research and corrections in the future. Text line 387 to 401. |
